# A dataset for estimating alfalfa leaf area and predicting leaf area index

**DOI:** 10.3389/fpls.2024.1290920

**Published:** 2024-02-13

**Authors:** Songtao Yang, Yongqi Ge, Jing Wang, Rui Liu, Daotong Tang, Ang Li, Zixin Zhu

**Affiliations:** College of Information Engineering, Ningxia University, Yinchuan, China

**Keywords:** leaf area estimation, leaf area index prediction, meteorological data, soil moisture data, alfalfa, deep learning

## 1 Introduction

Alfalfa (*Medicago sativa* L.) is the most widely planted perennial leguminous forage in the world and it plays a vital role in the diet of dairy cows (Fink et al., 2022; Zhang et al., 2023). Feeding alfalfa can substantially improve animal’s growth, reproduction, meat and milk production (Laroche et al., 2022; Ma et al., 2022; Motsinger, 2022). Alfalfa is currently the most commonly cultivated forage crop in China (Wan et al., 2022). Ningxia is located in the northwest agricultural and pastoral ecotone in China. It is one of the major alfalfa production areas in Northeast China. By 2022, the alfalfa planting area in Ningxia has reached 330000 hectares (Wang B. et al., 2022). The country’s aggressive promotion of “grain-forage substitution”, has expanded the planting area of silage maize, alfalfa and other feed crops, increased the harvest and storage capacity, promoted the specialization of feed varieties, production scale, and commercialization of sales, and opened up new possibilities for the Ningxia alfalfa business (Hui et al., 2021). However, the expanding cultivation area of alfalfa in Ningxia is facing significant challenges due to the influence of regional climate, limited water resources, and soil conditions (Qian et al., 2020). This has resulted in a growing contradiction between the increasing demand for water resources and the expanding area dedicated to alfalfa cultivation. Consequently, there is a growing need to monitor alfalfa yields to address yield gaps, including those caused by water stress and fertility limitations (Baral et al., 2022).

Timely and appropriate application of irrigation and fertilizer are essential for improving the yield and quality of alfalfa (Liu et al., 2021; Wang et al., 2021). The leaf area index (LAI) is a valuable dynamic indicator that reflects the size of crop populations and serves as a critical basis for analyzing crop growth patterns and predicting yield (Feng et al., 2022). It plays a crucial role in determining the net primary productivity, water and nutrient utilization, and carbon balance in alfalfa (Moghaddam and Mofidian, 2022). Therefore, it is important to study the characteristics and spatiotemporal changes of leaf area (LA) and LAI in alfalfa, which can improve yield estimation and the efficiency of water and fertilizer utilization in arid and semi-arid regions.

Currently, extensive research has been undertaken by researchers on the variations in LAI (Leaf Area Index) across different crops like rice, wheat, and maize. (Wang E. et al., 2022). Some of these studies have employed deep learning technology to estimate the LAI and assess its impact on crop yield (Jin et al., 2020; Qi et al., 2020; Tian et al., 2021). For example, Adeluyi et al. (2021) implemented rice experiments in the central northern region of Nigeria. Three irrigation treatments and three nitrogen fertilizer application levels were designed and LAIs were measured in each plot using the LAI-2200 plant canopy analyzer from December 2017 to April 2018. Zhang et al. (2021) conducted winter wheat experiments in the Xindian area of Luoyang City. They designed four nitrogen fertilizer treatments, and 132 LAI data points were collected in 2018 during the different growth stage of winter wheat. There are also studies on maize LAI experiments. Castro-Valdecantos et al. (2022) implemented maize breeding trials in Seville, Spain from 2018 to 2019. A total of 32 maize plots were selected, and two representative plants were randomly selected from each plot on each sampling date. The LAI for each experimental plot was estimated using the allometric growth relationship. However, the aforementioned literature did not publish the related datasets. In recent years, extensive research has been conducted on the growth characteristics, water consumption patterns, and irrigation regimes of alfalfa, with a focus on the interaction between field management practices, crop morphological characteristics, and yield (Liu et al., 2022; Ojaghlou et al., 2023; Wang et al., 2023). However, there have been relatively few studies on alfalfa LAI. To the best of our knowledge, there is currently no publication on alfalfa leaf area (LA) and LAI datasets in Northwest China, despite the fact that China is the second-largest producer of alfalfa in the world (Lv et al., 2023).

To address this issue, we proposed a dataset that can be utilized for estimating LA and predicting LAI of alfalfa. The dataset included meteorological data, soil moisture measurements at multiple depths (0-10 cm, >10-20 cm, and >20-30 cm), and various parameters associated with alfalfa growth, including plant height, LAI, yield, and leaf dimensions (length, width, and area). The main objective of this research work is to provide a dataset of the characteristics and spatiotemporal changes of LA and LAI in alfalfa for estimating yield and optimizing field water and fertilizer management.

## 2 Value of the data

(1) The dataset provides growth data of alfalfa under different water and nitrogen treatments. A total of 5118 alfalfa samples were collected, and the length, width, and LA of 76688 leaves were measured. Moreover, the LAI of different experimental plots was calculated. This dataset filled the gap in publicly published alfalfa growth data in northwest China, helping to address the challenges of estimating LA and predicting LAI of alfalfa in different production environments. Furthermore, the dataset provides essential data support for optimizing water and nitrogen strategies in alfalfa field management.

(2) The dataset includes meteorological data throughout the entire growth period of alfalfa and soil moisture data at various depths. Integrating multiple features is beneficial for expanding the applicability of estimation and prediction models and improving their accuracy.

## 3 Materials and methods

### 3.1 Experimental design

In this study, the interest area is the Ningxia Irrigation Area of Yellow River (NIR), which is situated in the Ningxia Hui Autonomous Region in China. The experiment was implemented at the first grassland experiment site of Ningxia State Farm Maosheng Prataculture Co., Ltd., located on the eastern foothills of the Helan Mountains in the alluvial fan plain. The effects of different water and nitrogen application amounts on alfalfa LA and LAI vary, and the responses of different alfalfa varieties to LA and LAI also differ under rates of irrigation and nitrogen application. Based on the actual cultivation of alfalfa in the NIR, we designed two experiments to collect LA and LAI data for alfalfa (2017-2018 and 2022). During the period from 2019 to 2021, the experimental field was utilized for planting silage maize using a rotation method.

The first experiment, conducted from 2017 to 2018, involved collecting data on the same alfalfa variety, Juneng 7, under different water and nitrogen treatments. Juneng 7 is a widely planted alfalfa variety in Ningxia. The seeding rate was 22.5 kg ha^-1^, with a seeding depth of 2 cm and row spacing of 15 cm. According to the irrigation methods used in the NIR, we designed two irrigation methods: flood irrigation and subsurface drip irrigation. The experiment adopted a split-plot design, with irrigation amount as the main treatment and nitrogen application amount as the sub-treatment. 1) A total of 5 levels were designed for the irrigation amounts, including 1199 mm (flood irrigation plot, 12000 m^3^ ha^-1^), 525 mm (W1, 5250 m^3^ ha^-1^), 600 mm (W2, 6000 m^3^ ha^-1^), 675 mm (W3, 6750 m^3^ ha^-1^), and 750 mm (W4, 7500 m^3^ ha^-1^), respectively. 2) A total of 4 levels were designed for the nitrogen application rates, including N0 (0 kg ha^-1^), N1 (60 kg ha^-1^), N2 (120 kg ha^-1^), and N3 (180 kg ha^-1^), respectively. This experiment consisted of 18 treatments, each repeated 3 times. The irrigation schedule was implemented when the alfalfa was turning green. The specific irrigation schedule is shown in **Table 1**.

**Table 1:** Irrigation schedules during the entire growth period of alfalfa in 2017-2018 and 2022.

Cutting time	Irrigation date	Growth stage	Irrigation amount (mm)				
			W0	W1	W2	W3	W4
First cutting	2017-04-02 2018-04-20	Vegetative stage	180	52	60	67	67
	2017-04-23	Bud stage	—	45	52	52	67
	2018-05-03						
	2017-05-12 2018-05-14	Bud stage	180	37	45	52	52
	2017-05-20 2018-05-22	Early flowering stage	—	37	37	45	52
Second cutting	2017-06-03 2018-06-13	Regrowth	150	45	52	52	60
	2022-07-08		—	45	52	60	—
	2017-06-09 2018-07-06	Bud stage	150	37	45	52	52
	2022-07-19		—	45	45	52	—
	2017-06-21 2018-07-16	Early flowering stage	—	37	37	45	52
	2022-07-29		—	37	45	52	—
Third cutting	2017-07-04 2018-07-16	Regrowth	210	45	52	52	60
	2017-07-14 2018-07-30	Bud stage	—	37	37	45	52
	2022-08-11		—	37	45	45	—
	2017-07-24 2018-08-07	Early flowering stage	—	30	37	45	45
	2022-09-02		—	45	52	60	—
Fourth cutting	2017-08-07 2018-08-14	Bud stage	150	37	45	52	60
	2017-09-07 2018-09-07	Early flowering stage	—	37	45	52	60
Total			1020	476	544	611	679

In the second harvest of 2017, irrigation during the regrowth period was temporarily canceled. W0 represents flood irrigation, while the others represent subsurface drip irrigation. In 2022, which is the first planting year of alfalfa, the forage is harvested in three cutting. To ensure a good rate of emergence, irrigation was conducted randomly based on the soil moisture status and no specific irrigation treatments were formulated for the first cutting.

The second experiment was conducted in 2022, where data was measured on seven different alfalfa varieties: Juneng No.7, Zhongmu No.3, Gannong No.4, Zhonglan No.2, DF310, Miracle, and Algangjin. This experiment focused on measuring alfalfa growth data under different rate of irrigation while keeping the nitrogen application rate constant. The alfalfa was sown on April 16, 2022, with a seeding rate of 22.5 kg ha^-1^, a seeding depth of 2 cm, and a row spacing of 15 cm. The irrigation method used in this experiment was surface micro-sprinkler irrigation, with three irrigation treatments: 525 mm (W1, 5250 m^3^ ha^-1^), 675 mm (W2, 6750 m^3^ ha^-1^), and 750 mm (W3, 7500 m^3^ ha^-1^). The irrigation schedule can be seen in **Table 1**.

### 3.2 Data acquisition

#### 32.1 Alfalfa growth data acquisition

**Figure 1:**
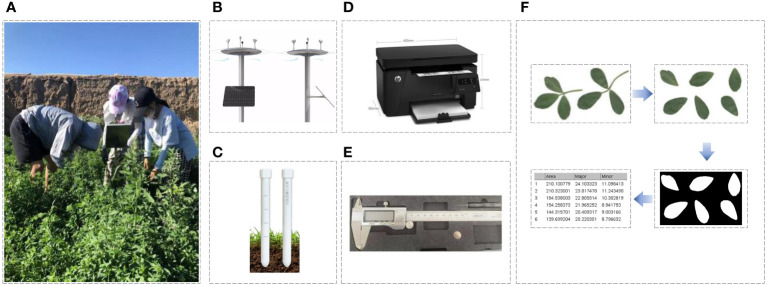
The experimental scenario and equipment deployed. **(A)** Data collection scenario; **(B)** Meteorological stations; **(C)** soil moisture sensors; **(D)** Scanner; **(E)** vernier caliper; **(F)** Leaf area measurement process.

**Figure 2:**
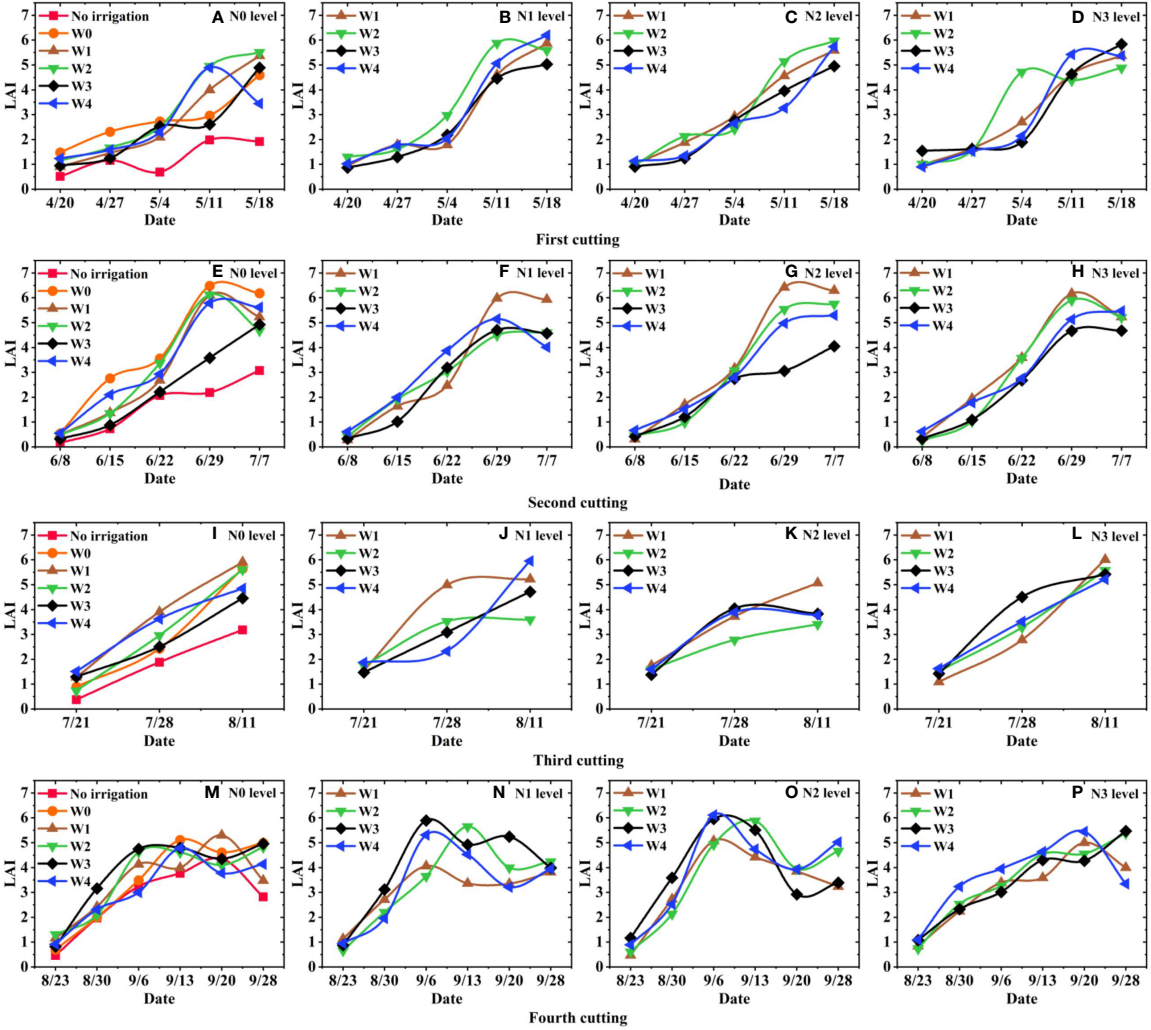
The LAI of alfalfa under different water and nitrogen treatments in 2018. **(A, E, I, M)** represents the LAI at N0, **(B, F, J, N)** represents the LAI at N1, **(C, G, K, O)** represent the LAI at N2, and **(D, H, L, P)** represents the LAI at N3.


In this study, three random plants were selected from each plot for measurement after each cutting of alfalfa regrowth (**Figure 1A**). To eliminate measurement errors caused by water loss, stem diameter, plant height, leaf length, leaf width, and leaf area were all measured on the same day of sampling. Additionally, the number of leaves was counted. The measurement methods differed slightly between 2017-2018 and 2022. (i) In 2017-2018, we randomly selected intact and healthy leaves in layers, with 6 leaves selected from each layer. Leaf length and leaf width were measured using a vernier caliper with an accuracy of 0.01 mm (**Figure 1E**). The leaf area was calculated using the leaf area estimation formula (Faverjon et al., 2017). The specific calculation formula is shown in Equation (1). (ii) In 2022, we used an HP M126a scanner (**Figure 1D**) and Image J software to measure leaf length, leaf width, and leaf area (Sun et al., 2015). In this method, the leaves were flattened and placed on a scanner bed (HP M126a) and scanned sequentially at a resolution of 300 dpi, with the images saved in jpg format. Since the stems were not removed during scanning, the stem portion was manually removed using the drawing tool in Image J software before calculating the leaf area (**Figure 1F**). For leaf area measurement, the color mode was changed to 8-bit grayscale, and the “Size” value in the “Analyze Particles” function was set to 20-Infinity, considering areas below 20 mm² as impurities and excluding them from area recognition. The calculation method of LAI is shown in Equation (2) (Gower et al., 1999). **Figure 2** shows the LAI of alfalfa under different water and nitrogen treatments in 2018.

(1) LA=K×L×W

Where *LA* represents the leaf area; *L* and *W* represent the leaf length and the leaf width (mm), respectively; and *K* represents the correction coefficient.


(2) LAI=AVGLA×ρ/S

Where *LAI* represents the leaf area index, *AVGLA* is the average leaf area per plant, *ρ* is the planting density, and *S* is the planting area.

#### 32.2 Yield data acquisition

Yield refers to the total biomass of a crop per unit of land area, excluding the root system. It reflects alfalfa productivity, and a higher yield signifies greater productivity. It is directly influenced by meteorological circumstances as well as diverse water and nitrogen management strategies. During the growth period of the early flowering stage, we carefully selected alfalfa plants with uniform and consistent growth for harvest. Use the diagonal method to take 3 sample areas in each plot, with each sample area measuring 1 m^2^. The stubble height was maintained 5 cm. We measured the fresh yield of alfalfa after removing other weeds. Additionally, we took about 300 g of fresh alfalfa samples back to the laboratory and let them naturally dry in the wind until they reach below 20% moisture content. Then, we estimated forage dry matter yield.

#### 32.3 Meteorological and soil data acquisition

In this study, we deployed the ET007 Tianqi micro meteorological station (**Figure 1B**) and the ET40 intelligent soil moisture sensor (**Figure 1C**), which are specialized equipment designed for scientific research, to collect environmental and soil data.

Meteorological data were collected using Campbell Scientific data recorders, with a measurement interval of 60 seconds and data output of every 30 minutes. The weather station recorded various parameters including temperature (°C), net radiation (MJ m^-2^), CO_2_ concentration (mg m^-3^), soil moisture (m^3^ m^-3^), relative humidity (%), wind direction and wind speed (m s^-1^). According to the records of the meteorological station, the average temperatures during the alfalfa growth period measured in the experimental area were 20.6°C, 20.2°C, and 23.0°C in 2017, 2018, and 2022, respectively. The daily average temperature and effective accumulated temperature were estimated using Python 3.6 and the PyCharm program (Linge and Langtangen, 2020).

Soil moisture sensors were deployed at depths of 0-10 cm, >10-20 cm, and >20-30 cm with a data collection interval of 10 minutes for real-time monitoring over 24 hours. Due to limited experimental conditions, soil moisture was measured in seven different treatments in 2017-2018. These treatments included W2N0, W2N1, W2N2 and W2N3 under the same irrigation amount, as well as W1N2, W3N2 and W4N2 under the same nitrogen application rate.

## 4 Description and analysis of dataset

The dataset contains a total of five files. The dataset is fairly straightforward for setting up because we put data in files that correspond to the proper annotations. The dataset provides growth data of alfalfa under different water and nitrogen treatments. A total of 5118 alfalfa samples were collected, and the length, width, and LA of 76688 leaves were measured. The detailed information of the dataset is shown in **Table 2**.

**Table 2:** Summary of five parts of the dataset.

Dataset	Folder name	Data indicators	Sample size
Leaf area index data	LAI data	Leaf area index	955
		Plant height	968
		Stem diameter	839
Leaf area data	Leaf area data	Leaf Length	76688
		Leaf width	
		Leaf area	
Meteorological data	Meteorological data	Circumstance temperature	613
		Dew point temperature	
		Environmental humidity	
		CO_2_ concentration	
		Wind speed	
		Wind direction	
		Ultraviolet radiation	
		Effective accumulated temperature	476
Soil moisture data	Soil Moisture data	Soil moisture	10668
Yield data	Yield data	Yield	173

LAI is a key indicator for evaluating the growth structure of individual alfalfa plants or entire populations (Hammond et al., 2023). This study used Origin software to analyze the effects of different water regimes and nitrogen rates on the leaf area index of Juneng 7 alfalfa in 2018. **Figure 2** shows the dynamic trend of alfalfa LAI under different water and nitrogen treatments in 2018 of the datasets. **Figure 2** shows that the overall trend of LAI changes in alfalfa is essentially consistent with that of alfalfa harvested for forage purposes. After the plant turned green, alfalfa grew rapidly, and the LAI increased rapidly as the temperature rose. Due to influence of temperature during the growth period, the LAI of the second and third cuttings rapidly increased after regrowth stage, while the LAI of the first and fourth cuttings showed slower growth (**Figure 2**). The LAI of the fourth cutting exhibited a declining trend in the later growth period, which aligned with Bai and Bao (2002)’s findings that as autumn approaches, the LAI of alfalfa typically decreases during the later development stage (after the early flowering stage) (**Figures 2M-P**). Additionally, the maximum LAI of the first, second, and third cuttings can reach or approach 6, whereas the maximum LAI of the fourth cutting in the observed region was around 5 (**Figure 2**). This result consistent with Gao and Hannaway (1985)’s research who stated that the maximum LAI of the fourth cutting indicates that unpredictable climate factors affect alfalfa growth during autumn.

Moreover, the growth of alfalfa is influenced and restricted by water and nitrogen. Additionally, previous research has shown that water has a greater impact on alfalfa yield compared to nitrogen (Sun et al., 2005). Through analyzing the dataset, it was found that different irrigation treatments have different effects on the alfalfa LAI (**Figure 2**). The overall performance of alfalfa LAI under W2 irrigation treatment was higher compared to other treatments, indicating that an appropriate amount of irrigation can impact the stem-leaf ratio of alfalfa and consequently affect the crude protein content of alfalfa (Su et al., 2011). However, under the same irrigation treatment, the impact of different nitrogen application rates on alfalfa LAI was not observed significant. Compared to other nitrogen treatments, the LAI performance under low nitrogen application rates was better. Upon analyzing the dataset, we observed that the response of LAI to nitrogen application varied across different alfalfa planting years (**Figure 2**). Notably, 2018 marked the third year of alfalfa planting. Based on the analysis of **Figure 2**, it is found that the impact of different nitrogen application rates on alfalfa LAI is not significant. This finding aligned with the research conclusion that applying nitrogen fertilizer during the year of alfalfa planting can improve plant growth and enhance winter survival. However, after more than two years of planting, alfalfa can rely on its own rhizobia nitrogen fixation to meet its nitrogen needs (Peters and Stritzke, 1970; Eardly et al., 1985; Gao et al., 2020). Additionally, significant variations were observed in the response of LAI to water and nitrogen across different alfalfa varieties and cuttings.

## 5 Potential use

The dataset holds significant practical importance within the context of deep learning advancements. It has the potential to assist in establishing models for estimating alfalfa leaf area and predicting leaf area index in complex physical environments. Furthermore, this dataset provides vital data support for alfalfa yield estimation and optimization of water and nitrogen strategy in field management.

## Data availability statement

The datasets presented in this study can be found in online repositories. The names of the repository/repositories and accession number(s) can be found below: https://github.com/im1robot/leaf-area-and-leaf-area-index.git.

## Author contributions

SY: Data curation, Writing – original draft, Writing – review & editing. YG: Data curation, Funding acquisition, Investigation, Writing – review & editing. JW: Supervision, Writing – review & editing. RL: Data curation, Supervision, Writing – review & editing. DT: Supervision, Writing – review & editing. AL: Supervision, Writing – review & editing. ZZ: Supervision, Writing – review & editing.
